# Depressive symptoms are associated with a functional polymorphism in a miR-433 binding site in the *FGF20* gene

**DOI:** 10.1186/s13041-018-0397-0

**Published:** 2018-09-21

**Authors:** Karen M. Jiménez, Angela J. Pereira-Morales, Ana Adan, Sandra Lopez-Leon, Diego A. Forero

**Affiliations:** 1 Laboratory of Neuropsychiatric Genetics, Biomedical Sciences Research Group, School of Medicine, Universidad Antonio Nariño, 110231 Bogotá, Colombia; 2 Department of Clinical Psychology and Psychobiology, School of Psychology, University of Barcelona, Barcelona, Spain; 3 Institute of Neurosciences, University of Barcelona, Barcelona, Spain; 4 Novartis Pharmaceuticals Corporation, One Health Plaza, East Hanover, NJ 07936-1080 USA

**Keywords:** Depressive symptoms, Mental health, MicroRNAs, Dopaminergic genes

## Abstract

Genetic studies of major depressive disorder and its associated endophenotypes are useful for the identification of candidate genes. In recent years, variations in non-coding RNA genes, such as miRNAs, have been explored as novel candidates for psychiatric disorders and related endophenotypes. The aim of the present study was to evaluate the possible association between a functional polymorphism (rs12720208) in the *FGF20* gene, which regulates its modulation by miR-433, and depressive symptoms in young adults. A sample of 270 participants from Colombia were evaluated with the Hospital Anxiety and Depression Scale - Depression Subscale (HADS-D) and genotyped for the rs12720208 polymorphism using a TaqMan assay. A lineal regression analysis was used. A statistically significant association of the functional polymorphism in the *FGF20* gene (rs12720208) with depressive symptoms was found. It was observed that individuals with the G/A genotype had higher scores for the HADS-D subscale. Our results are the first description in the scientific literature about a significant association between a functional polymorphism in the FGF20 gene, which regulates its modulation by miR-433, and depressive symptoms.

## Introduction

Major depressive disorder (MDD) leads to major personal, physical and social consequences, affecting more than 350 million people worldwide [1]. Although a large number of studies on depression have been carried out, leading to a significant progress in understanding this psychiatric disorder, there is the need for more studies to find its molecular risk factors and pathophysiological mechanisms [2]. Genetic studies of MDD and its associated endophenotypes are useful for the identification of candidate genes [2]. Depressive symptoms are interesting endophenotypes for MDD that have been used in genetic studies [3]. In recent years, variations in non-coding RNA genes, such as miRNAs, have been explored as novel candidates for psychiatric disorders and related endophenotypes [4].

Polymorphisms in genes that are involved in the dopaminergic systems have been postulated as novel candidates for MDD, considering the role of dopamine in neural processed related to motivation and reward [2]. The *FGF20* gene is a member of the FGF (fibroblast growth factors) family, composed by 22 members, and it is located on the 8p21.3 chromosome [5]. Fibroblast growth factors are polypeptides involved in neural functions and development; *FGF20* gene specifically has been associated with the development and function of dopaminergic neurons and it is expressed in prefrontal cortex and cingulate cortex [6]. In the 3′ untranslated region (UTR) of this gene, it has been shown that a functional polymorphism (rs12720208) disrupts a binding site for miR-433, generating an increase in translation of *FGF* [7]. A study found that the rs12720208 polymorphism in a sample of young adults was associated with low scores in verbal episodic memory in A allele carriers [8]. In addition, they found that the rs12720208 polymorphism can alter FGF20 expression and have effects on hippocampal morphology [8].

*FGF20* gene has been associated with psychiatric disorders such as schizophrenia, attention-deficit and hyperactivity disorder and it has been reported a dysregulation of the FGF system in depressive disorder [6]. This relationship is explained in part by the hypothesis of the role of neurogenesis in depression; in this context, *FGF* has been linked to favoring neurogenesis and in humans FGF action has been involved in the stress response [6]. Furthermore, some reports have shown an increase in FGF receptor expression with selective serotonin reuptake inhibitors treatment in depressive subjects [9]. However, to date no published study have reported the association between *FGF20* gene and depression; although some studies have reported association between another FGF system transcripts, such as FGF2, FGF12 and FGFR2 with major depressive disorder [9].

The aim of the current study was to evaluate the possible association between the functional polymorphism in the *FGF20* gene and depressive symptoms in young adults.

## Methods

### Participants

The current study analyzed a sample of 270 young adults, living in Bogotá, Colombia. Participants were at least 18 years or older, were unrelated and did not have personal history of neuropsychiatric disorders, according to self-report. The subjects had a mean age of 21.3 years (SD = 3.8) and 75.1% were women. The socioeconomic status (SES) of the total sample was represented mainly by low (33.5%) and medium (44.5%) strata, according to self-report. All participants signed a written informed consent and the study was approved by the institutional ethics committee (Universidad Antonio Nariño).

### Assessment of depressive symptoms

To assess the presence of depressive symptoms, the depression sub-scale (HADS-D) of the Hospital Anxiety and Depression Scale (HADS) was used. This subscale has 7 Likert-type items related with common cognitive and emotional symptoms of depression, mainly anhedonia and it has been extensively used in primary care and clinical research [10]. It has been validated in Spanish and in a Colombian sample [11].

### Genotyping

A salting out method was used for the extraction of genomic DNA from 400 μl of peripheral blood. DNA quantification was carried out in a Qubit 2.0 fluorometer (Thermo Fisher Scientific, MA, USA) using the Qubit dsDNA BR assay kit (Thermo Fisher Scientific) and then normalized to 10 ng/μl and stored at 4 °C until used. Genotyping of the rs12720208 SNP in the *FGF20* gene was made using the TaqMan SNP genotyping assay (Thermo Fisher Scientific). For the qPCR reaction, it was used 5 μl of TaqMan genotyping Master Mix (Thermo Fisher Scientific), 0.5 μl of the pre-design TaqMan probe (c__31674955_10, Thermo Fisher Scientific), 20 ng of genomic DNA and ultra-pure water, for a final volume of 10 μl. The standardized PCR program for TaqMan assays was performed in a CFX96 Touch Real-Time PCR system (BioRad, Hercules, CA, USA). Genotyping results were analyzed with the CFX Manager Software v.3.0 (BioRad). A random 10 % of the samples was reanalyzed for genotyping confirmation.

### Statistical analysis

To assess the normal distributions of the HADS-D score, an analysis of skewness and kurtosis was used. In addition, the psychometric analysis of the HADS-D was performed, using Cronbach’s alpha for internal consistency. These statistical analyses were conducted with the Statistical Package for the Social Sciences (SPSS v. 18). Allelic and genotype frequencies, Hardy-Weinberg equilibrium and the analysis of a possible association of the genotypes with the HADS-D subscale scores were calculated using the SNPStats program [12]. This program uses a linear regression model, which was adjusted by age and gender. The results of this linear regression model were summarized by the mean and the standard errors of the compared genotypes showing a *p* value for  the differences [12].

## Results

In the current sample, we found a Cronbach’s alpha of 0.65 for HADS-D. The most frequent genotype was the G/G, followed by the G/A genotype (91% and 9%, respectively). The A allele was found in 5% and the G allele was found in 95% of the sample analyzed. We did not find the A/A genotype. Our sample was in Hardy Weinberg equilibrium (*p* = 1).

**Fig. 1:**
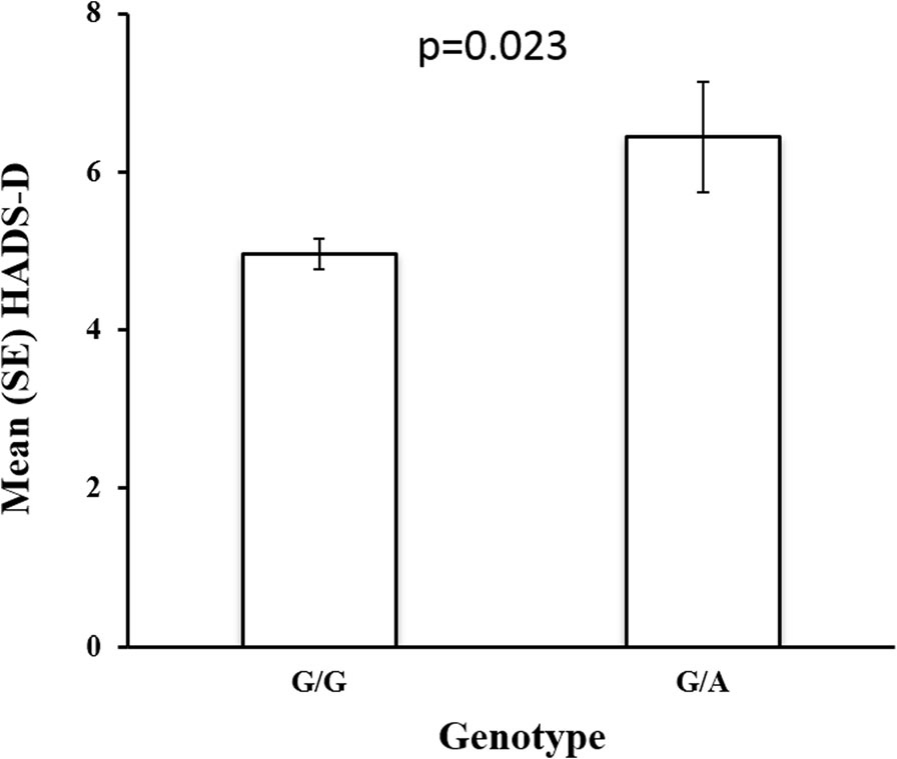
Association of the functional polymorphism rs12720208 in the *FGF20* gene and depressive symptoms in a Colombian sample

A statistically significant association of the functional polymorphism in the *FGF20* gene (rs12720208) with depressive symptoms was found (Fig. 1).The mean of individuals with the G/G genotype was 4.96 (SE 0.2) and of individuals with the genotype G/A 6.44 (SE 0.7). It was observed that individuals with the G/A genotype had higher scores for the HADS-D subscale (Fig. 1).

## Discussion

There is a need for the identification of additional genetic risk factors for MDD [2]. In this context, the genetic exploration of endophenotypes for MDD and the study of novel pathways and mechanisms, such as the analysis of polymorphisms in miRNA binding sites in genes involved in dopaminergic function, might be useful [4]. The members of the *FGF* family are potential candidates for MDD, considering previous studies that have shown an association between growth factor levels and hippocampal volume, taking into account that hippocampal volume has been negatively correlated with susceptibility to stress-induced disease [9], such as anxiety disorders, including posttraumatic stress disorder.

*FGF20* gene has been reported as having neurotrophic properties associated with the survival of dopaminergic neurons [13]. The results of the current study showed that young subjects with G/A genotype of the rs12720208 polymorphism have higher depressive symptoms compared with G/G carriers. This is concordant with previous studies about lower hippocampal volume in depressive patients. In a meta-analytic study with 434 depressive subjects and 379 controls (mean ages from 28.4 to 74.3) it was found that depressive patients had lower hippocampal volume compared with controls [14]. Additional studies reported that smaller hippocampus volumes have been associated with severity of depressive symptoms, poor response to treatment, age at onset of disease and history of childhood abuse [1]. Importantly, several studies have described that the hippocampus is largely susceptible to life adversities and stress, being a mediator of the relationship between adversities in early-life and depression [1]. These results could be correlated, in a future research neuroimaging study that would be carried out  in individuals who showed high scores in the HADS-D subscale, since previous findings have shown that A allele carriers had a decrease in hippocampal volume [8]. Given that the studies with low mean ages included in the meta-analyses did not show significant hippocampal volume differences [14], age needs to be taken into account in future studies.

Limitations of the study included a convenience sample design, which includes a higher proportion of females. In addition, it was not possible to control for factors that could be confounding, such as stress, life adversities and other comorbidities. Further studies in different populations (other ethnicities) and in patients with major depressive disorder are needed to replicate this finding.

## Conclusion

In conclusion, we found that the functional polymorphism rs12720208 in the *FGF20* gene, which regulates its modulation by miR-433, is associated with depressive symptoms in young adults. Our results are the first description in the scientific literature that found this significant association and can be understood in the light of the previous reports showing alterations in *FGF* system in patients with affective disorders, including MDD [1].

## Abbreviations

FGF20: Fibroblast growth factor 20
HADS: Hospital Anxiety and Depression Scale
MDD: Major depressive disorder
SD: Standard Deviation
SES: Socioeconomic status
SPSS: Statistical Package for the Social Science
